# Supplementary material for: O/W Pickering Emulsions Stabilized with Cellulose Nanofibrils Produced through Different Mechanical Treatments
Source: Foods. 2021 Aug 15;10(8):1886. doi: 10.3390/foods10081886 (PMC8394195; doi:10.3390/foods10081886)
Supplement: Supplementary file 1 [file foods-10-01886-s001.zip › foods-1313965-supplementary.pdf]

S.1 HPH treatment on tomato pomace

The effect of HPH treatment on the rupture of cell walls and membranes is evident from the following micrograph. In particular, as the treatment time increases, the dimensions of tomato pomace particles decrease and occurs the formation of filamenous debris.

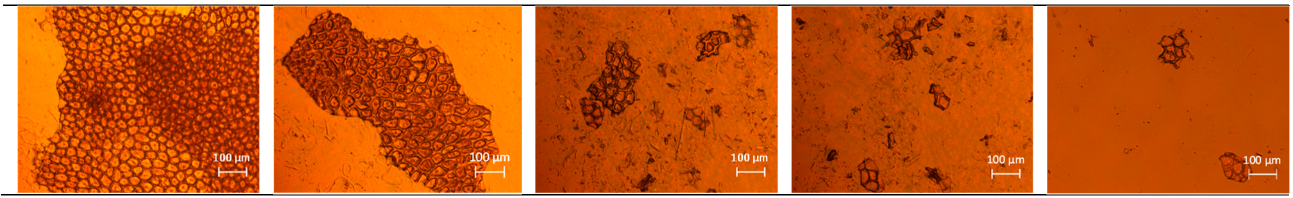

Figure S1. Micrographs of HPH-treated tomato pomace as a function of treatment time.

S.2. Emulsion stability

The change of emulsion rheological properties over time provides important pieces of information for interpreting storage stability, as the increase of viscosity could retard droplets movement and delay the creaming process (Fernández Sosa et al., 2021). Table S1 shows that the viscosity of all emulsions is lower in acidic conditions, which may be due to the weak viscoelasticity of the interfacial layer. Moreover, the viscosity of all emulsions decreased with increasing storage time. Only HPH-NC stabilized emulsion is not significantly affected by the storage; this effect could be ascribed to the stronger interaction between molecules presented.

HPH-NC and HPH-TP exhibited the highest viscosity among all the emulsions, regardless of the storage and pH conditions. Based on the aforementioned discussion about the microstructure analysis, the enhanced viscosity was beneficial to increase the stability of emulsions stabilized with HPH-NC and HPH-TP, with better resistance to droplet aggregation over the entire storage and centrifugal forces.

Table S1. Viscosity values for each emulsion as a function of storage period and pH environmental conditions.

pH =2.0		Viscosity (Pa·s)			
		BM30-CP	BM60-CP	HPH-NC	HPH-TP
Storage period (day)	0	0.032 ± 0.001^{c}	0.023 ± 0.001^{b}	14.820 ± 0.940^{a}	1.548 ± 0.214^{c}
	28	0.024 ± 0.001^{b}	0.015 ± 0.002^{a}	14.293 ± 0.446^{a}	1.337 ± 0.068^{bc}
Centrifugation at 3,500 rpm	10 min	0.023 ± 0.003^{b}	0.018 ± 0.002^{a}	14.502 ± 0.314^{a}	1.261 ± 0.017^{b}
	20 min	0.019 ± 0.001^{a}	0.015 ± 0.003^{a}	14.404 ± 0.142^{a}	0.836 ± 0.066^{a}
**pH =7.0**		**Viscosity (Pa·s)**			
		BM30-CP	BM60-CP	HPH-NC	HPH-TP
Storage period (day)	0	0.381 ± 0.006^{d}	0.069 ± 0.002^{c}	21.138 ± 0.889^{a}	1.015 ± 0.148^{b}
	28	0.182 ± 0.009^{c}	0.049 ± 0.001^{b}	20.053 ± 0.487^{a}	0.819 ± 0.014^{ab}
Centrifugation at 3,500 rpm	10 min	0.157 ± 0.002^{b}	0.026 ± 0.006^{a}	18.623 ± 1.743^{a}	0.747 ± 0.071^{a}
	20 min	0.138 ± 0.002^{a}	0.018 ± 0.003^{a}	17.733 ± 2.020^{a}	0.739 ± 0.144^{a}

pH =12.0		Viscosity (Pa·s)			
		BM30-CP	BM60-CP	HPH-NC	HPH-TP
Storage period (day)	0	0.043 ± 0.001^{c}	0.029 ± 0.002^{c}	6.858 ± 0.104^{b}	0.615 ± 0.034^{b}
	28	0.025 ± 0.001^{b}	0.019 ± 0.001^{b}	4.902 ± 1.434^{a}	0.309 ± 0.021^{ab}
Centrifugation at 3,500 rpm	10 min	0.020 ± 0.002^{a}	0.020 ± 0.001^{b}	4.333 ± 0.305^{a}	0.203 ± 0.023^{a}
	20 min	0.018 ± 0.002^{a}	0.015 ± 0.002^{a}	4.334 ± 0.298^{a}	0.129 ± 0.064^{a}

The letters indicate significant differences ($p < 0.05$) for the same sample during the storage period ($n = 5$).

Therefore, HPH-NC emulsion with higher viscosity prevents mobilization of the continuous phase between the oil droplets, resulting in a more stable emulsion. Additionally, microscopic inspections were carried out to evaluate the stability of the prepared emulsions by observing their ability to resist physical changes under pH environmental factors after centrifugation at 35,000 rpm for 10 and 20 min. As seen from Figure S3, the oil in water emulsions prepared with all particles showed phase separation under acid condition. However, emulsions stabilized with HPH-NC and HPH-TP allowed for increased oil droplet stabilization.

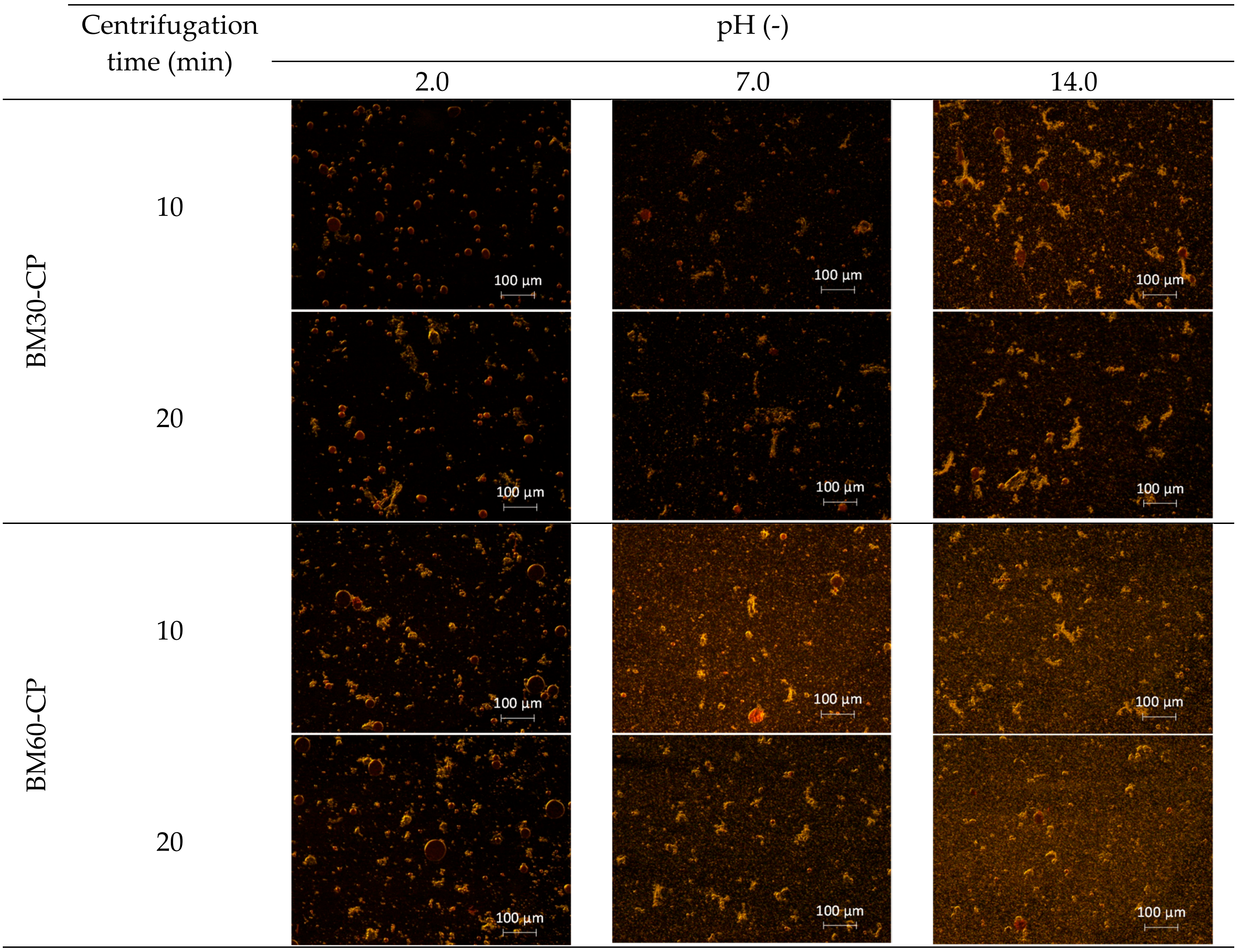

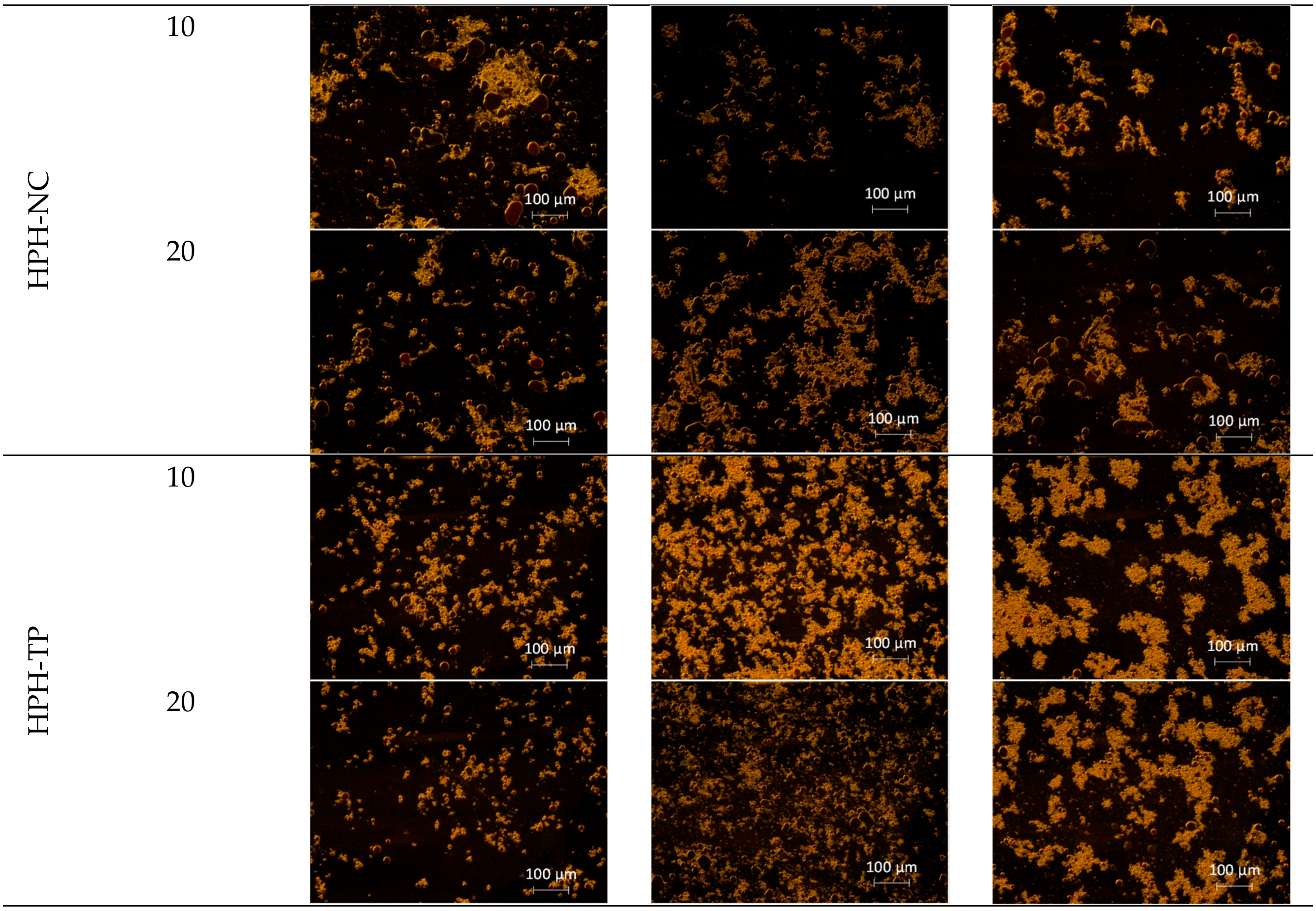

Figure S2. Fluorescence micrographs of emulsion stabilized with BM30-CP, BM60-CP, HPH-NC, and HPH-TP under different pH values during storage period at 4 °C.

Reference

Fernández Sosa, E.I., Chaves, M.G., Henao Ossa, J.S., Quiroga, A.V., Avanza, M.V., **2021**. Protein isolates from Cajanus cajan L. as surfactant for o:w emulsions: pH and ionic strength influence on protein structure and emulsion stability. Food Biosci. 42. https://doi.org/10.1016/j.fbio.2021.101159
